# Adapting artificial intelligence into the evolution of pharmaceutical sciences and publishing: Technological darwinism

**DOI:** 10.3389/jpps.2023.11349

**Published:** 2023-03-22

**Authors:** Neal M. Davies

**Affiliations:** Faculty of Pharmacy & Pharmaceutical Sciences, University of Alberta, Edmonton, AB, Canada

**Keywords:** artificial, intelligence, ChatGPT, evolution, pharmaceutical

“It is not the strongest of the species that survives, nor the most intelligent that survives. It is the one that is the most adaptable to change.”

-Charles Darwin

Evolution refers to the process by which species adapt to their environment and develop new traits over time. Similarly, technology has undergone significant changes over the years, with new innovations constantly emerging and rendering older technologies outdated. For instance, floppy disks used to be a popular way to store and transfer computer data before the advent of USB drives and cloud storage. Before the internet, encyclopedias were a popular means of accessing general knowledge and information. These examples illustrate how natural selection renders older technologies obsolete as new innovations emerge.

Scientific journals have also undergone significant evolution since the establishment of the first academic journals in the 17th century. Originally, scientific journals were published in print format. However, the rise of the World Wide Web enabled the Journal of Pharmacy and Pharmaceutical Sciences to provide articles freely available to the public, removing barriers to accessing scientific research *via* an “open access” path in 1998.

Pharmaceutical science is the field of science focused on the discovery, development, and manufacturing of drugs. Over the years, this field has undergone significant progress. Advancements in our understanding of the molecular and genetic basis of diseases have greatly contributed to this growth. Techniques such as combinatorial chemistry and computer modeling have immeasurably improved the efficiency of drug development. High-throughput screening allows scientists to quickly identify compounds with desired properties, and pharmacogenomics has led to the expansion of personalized medicine. Another example is the wide application of Network Pharmacology with a vast database to study commonly used traditional medicines in the evidence-based fashion.

The use of Artificial Intelligence (AI) will further facilitate pharmaceutical research and development. For example, the language models, such as Chat Generative Pre-Trained Transformer (GPT), which launched its public beta version in November 2022, has become increasingly prevalent in various scientific fields, including pharmaceutical sciences. These tools can be incredibly useful in assisting researchers and practitioners in their work, providing rapid access to a immense amount of information and aiding in data analysis and interpretation. However, it is essential to recognize their limitations and potential biases and use them appropriately.

In the pharmaceutical sciences, AI language models can be helpful in several ways. For example, they can assist in literature reviews, quickly summarizing extensive amounts of research and identifying key findings. They can also help in data analysis, modeling, and interpretation, providing insights into complex datasets that may be difficult to decipher manually. Additionally, they can assist in the maturing of drug discovery and development processes, aiding in the identification of potential drug targets and drug repurposing.

However, it is crucial to recognize that AI language models are not a substitute for human expertise and critical thinking. At the moment, these tools can only provide information based on the data they have been trained on, and their output is only as good as the quality of the data provided. Additionally, these models may have inherent biases based on the input data they have been trained on, which can lead to inaccurate or misleading results. Therefore, it is important to use AI language models in conjunction with human consciousness and experience.

Researchers and practitioners should carefully evaluate the output of these models, checking for accuracy and potential prejudices, and incorporate this information into their published work thoughtfully. Additionally, efforts should be made to improve the quality and diversity of the data used to train these models to reduce potential partiality and improve precision and accuracy. As AI language models continue to evolve and improve, it is likely that their use in the pharmaceutical sciences will become even more widespread. However, it is important to continue to evaluate and monitor their use to ensure that they are used ethically and effectively.

The administration of AI language models like ChatGPT in scientific publications is a growing trend, and while many journals may not have specific policies regarding their utility, they do have guidelines and policies that require rigor and transparency in research. This includes the application of computer programs and algorithms. Some products may be deemed by regulatory authorities as a medical device while others may be classified as Software of Unknown Provenance (SOUP). Authors should provide a detailed description of how the AI model was used, the data used to train the model, and the accuracy of the output. They must also disclose any potential biases or limitations of the AI model and acknowledge the role of human expertise and critical thinking in the research process. It is important to note that the praxis of AI language models in scientific research is an evolving field, and best practices and protocols are being developed. Therefore, authors should stay up-to-date with current research and guidelines and collaborate closely with journal editors and reviewers to ensure appropriate and transparent use of AI models.

Chat GPT AI is a remarkable example of the ongoing evolution of technology, a product of human ingenuity and the result of many years of development and refinement. It is an innovative tool that allows people to communicate, access, and process information in new ways. We must also recognize the current limitations of machine learning models which operate within certain pre-determined parameters and lack the ability to truly think or reason. AI models and programs are already useful and will undoubtedly continue to be essential tools in the pharmaceutical sciences. To maximize their potential, it is vital to ensure their appropriate and effective use in pharmaceutical research and practice. The use of AI in pharmaceutical sciences will be constantly changing, evolving, and adapting new traits, requiring researchers to stay updated with the latest advancements and best practices to adapt, survive and to succeed.

I wonder what Charles Darwin would think about all of this?

